# Rapid, progressive neuropathic arthropathy of the hip in a patient co-infected with human immunodeficiency virus, hepatitis C virus and tertiary syphilis: case report

**DOI:** 10.1186/1471-2334-11-159

**Published:** 2011-06-06

**Authors:** Lorenzo Drago, Elena De Vecchi, Marco Pasqualini, Laura Moneghini, Maurilio Bruno

**Affiliations:** 1 Laboratory of Clinical Chemistry and Microbiology, IRCCS Galeazzi Orthopaedic Institute, Milan, Italy; 2 Laboratory of Clinical Microbiology, Dept of Clinical Science L.Sacco, University of Milan, Milan, Italy; 3 Microsurgery Unit of Orthopaedic Dept, IRCCS Galeazzi Orthopaedic Institute, Milan, Italy; 4 U.O. Anatomia Patologica, Dept of Medicine, Surgery and Dentistry, University of Milan, San Paolo Hospital and Fondazione IRCCS Ospedale Maggiore Policlinico, Mangiagalli e Regina Elena, Milan, Italy

**Keywords:** Neuropathy, Syphilis, HIV, HCV, Charcot arthropathy

## Abstract

**Background:**

Syphilis is a chronic infection that is classified into three stages. In its tertiary stage, syphilis spreads to the brain, heart and other organs; the lesions may involve the skin, mucous membranes and bones. Neuropathic arthropathy associated with tertiary syphilis has rarely been described in Europe and its association with HIV-HCV co-infection has not been reported so far.

This article reports the case of a man with tertiary syphilis presenting with rapidly evolving neuropathic arthropathy of the hip and extensive bone destruction.

**Case presentation:**

On initial presentation, the patient complained of progressively worsening left-sided coxalgia without localized or generalized inflammation. The patient reported to have no history of previous infections, trauma or cancer. Plain x-ray films of the left coxofemoral joint showed marked degeneration with necrosis of the proximal epiphysis of femur and morphological alterations of the acetabulum without protrusion. Primary coxarthrosis was diagnosed and hip arthroplasty was offered, but the patient declined treatment. Three months later, the patient presented a marked deterioration of his general condition. He disclosed that he was seropositive for HCV and HIV, as confirmed by serology. Syphilis serology testing was also positive. A Girdlestone's procedure was performed and samples were collected for routine cultures for bacteria and acid fast bacilli, all resulting negative.

Although histological findings were inconclusive, confirmed positive serology for syphilis associated with progressive arthropathy was strongly suggestive of tertiary syphilis, probably exacerbated by HIV-HCV co-infection. The patient partially recovered the ability to walk.

**Conclusions:**

Due to the resurgence of syphilis, this disease should be considered as a possible cause of neuropathic arthropathy when other infectious causes have been ruled out, particularly in patients with HIV and/or HCV co-infection.

## Background

Tertiary syphilis can occur at any time two years after the primary infection, but the potential for reactivation continues for life. The spirochetes that seed most organs during primary and secondary syphilis typically remain inactive, held in check by the body's immune system; however, latent spirochetes can reactivate when immune control fails. Reactivation may occur when the immune defenses are weakened by cancer, AIDS, or other diseases, but usually no obvious explanation is apparent. The result is tertiary syphilis characterized by localized tissue destruction [1].

Syphilitic myelopathy is an exceedingly rare form of neurosyphilis, a complication of late or tertiary syphilis infection rarely associated with neuropathic or Charcot arthropathy sequela [2].

The etiology of Charcot arthropathy is uncertain. The first detailed description of the neuropathic aspect of the disease was given by Jean-Martin Charcot, whence the name, who noted this disease process as a complication of syphilis. Today, diabetes is considered the most common cause of Charcot arthropathy in the foot and ankle, whereas its occurrence in the hip is quite rare [3].

Several viral infections have been associated with osteoarticular disease [4-7]. Hepatitis C virus (HCV) is implicated in arthritis, myalgia, fibromyalgia and neuropathy [4-6]. Human immunodeficiency virus (HIV)-related musculoskeletal conditions include osteonecrosis, HIV-associated joint arthritis, and myopathies [7].

To our knowledge, neuropathic arthropathy associated with tertiary syphilis has seldom been described in Europe and no association between HIV-HCV co-infection, tertiary syphilis and this orthopaedic disease has been reported to date. Here we present a case of man with HIV-HCV co-infection and serology suggestive for tertiary syphilis with rapid, progressive destruction of the hip joint and bone resembling Charcot arthropathy. Surgical treatment according to Girdlestone's procedure (resection arthroplasty of the hip) is also described.

## Case presentation

A 69-year-old man was referred to the Microsurgery Unit of the Orthopaedic Department of the IRCCS Galeazzi Orthopaedic Institute in September 2009 because of left-sided coxalgia which had progressively worsened over the past year. Although the hip pain limited movement, he was still able to walk without the aid of crutches. There was no localized or generalized inflammation. Plain x-ray films showed marked degeneration of the left coxofemoral joint, with necrosis of the proximal epiphysis of femur and morphological alterations of the acetabulum without protrusion. The patient reportedly had no history of previous infections, trauma, or cancer; he was reluctant to give details on further questioning. No other investigations were performed and a clinical diagnosis of primary coxarthrosis was made. Hip arthroplasty was offered but the patient decided to postpone surgical treatment.

Three months later, he arrived at the Emergency Department of our institute because of a sudden deterioration of his condition and severe left hip pain. The patient was hospitalized.

**Figure 1:**
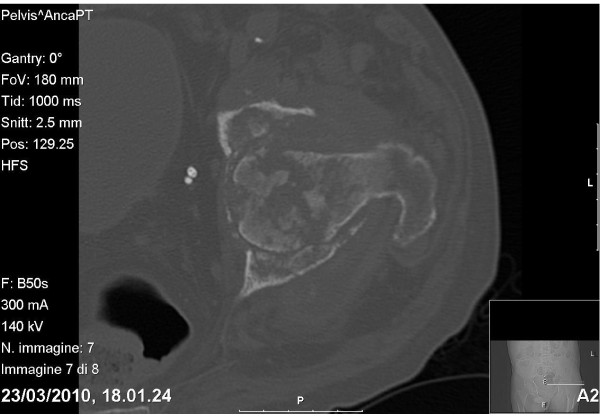
**CT image of the coxofemural joint showing extensive bone resorption**.

**Figure 2:**
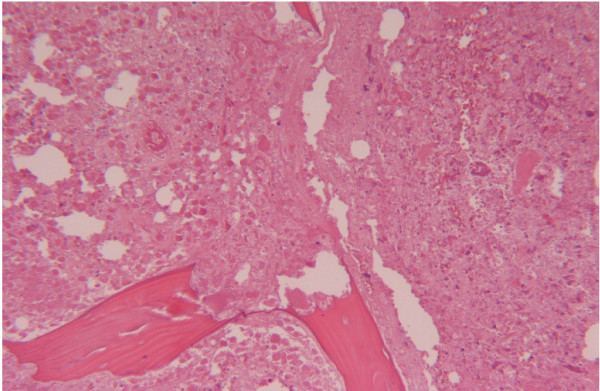
**Low-power photomicrograph showing extensive areas of bone resorption with few residual necrotic bony trabecules (Stain, hematoxylin and eosin; original magnification 40X)**.

The white cell count was elevated (10.1 × 10^9 ^cells/L; 74% neutrophils and 15.4% lymphocytes), the erythrocyte sedimentation rate (ESR) was 102 mm/h and the C reactive protein (CRP) level was 68.0 mg/L. The thromboplastin time was 21 sec (international normalized ratio [INR] 3.0); the activated partial thromboplastin time was 1.68 sec. CT scans revealed a wide zone of bone destruction (Figure 1). Because of poor bone quality and high risk of infection, a Girdlestone's procedure (non-prosthetic arthroplasty) was performed with a view to delay definitive prosthesis until the patient's general condition had improved. Histologic examination revealed complete destruction of the bone structure, with extensive areas of bone resorption and very few residual necrotic bony trabecules (Figure 2). The intertrabecular spaces revealed coagulative necrosis with histiocytic infiltration. In the residual vital bone there was lymphoplasmacellular infiltration, with non-necrotizing granulomas and multinucleated giant cells. Special staining (PAS, Ziehl-Neelsen, and Warthin-Star) showed no evidence of microorganisms.

Microbiological cultures of surgical samples for bacteria, acid-fast bacilli and fungi were negative. Since no apparent infectious etiology could be determined, serological syphilis testing was performed. The serum rapid plasma reagin test was positive; the *Treponema pallidum *particle agglutination test (TP-PA) was positive at a titer of 1: 20480, indicating reactivation of syphilis. The TP-PA titer was confirmed on a second independent sample obtained two days later.

The rates of syphilis have been increasing in the HIV-seropositive population. This man did not voluntarily reported his HIV or HCV serostatus, but the yield from questions about sexual history can be quite low. Only when confronted with the laboratory findings did he disclose his true serostatus. A diagnosis of HIV-HCV co-infection was subsequently confirmed by serology.

According to test results obtained from another laboratory in January 2010, the HIV viral load was below the limit of detection (40 IU/mL) due to previous successful antiretroviral therapy; the HCV RNA was 1071650 IU/mL; the liver enzyme values were as follows: GOT 40 U/L; GPT 54 U/L; and γGT 251 U/L. On admission to our hospital, the liver enzyme values were: GOT 52 U/L; GPT 67 U/L; and γGT 445 U/L. The man stated that he was not in therapy for HIV or HCV infection. No other data about his clinical history could be obtained because he had been treated in another hospital.

Further serological testing excluded recent infection by Epstein-Barr Virus (EBV) and cytomegalovirus (CMV), although high IgG levels were detected for both viruses.

The interferon-gamma release assay (Quantiferon TB-Gold) was negative; latent or active mycobacterial infections were therefore excluded. Although histological findings were inconclusive, when we considered the constellation of findings from surgical specimen culture and chest imaging and the absence of clinical signs or symptoms of active tubercular infection, the positive serology for syphilis associated with a progressive arthropathy was thought to be diagnostic of tertiary syphilis for which no treatment was given.

On the basis of these findings, the diagnosis was revised to neuropathic destructive arthropathy of the left hip.

After an immobilization period of 20 days, the patient entered a physical rehabilitation program which is still continuing. He has partially recovered weight-bearing but still requires the aid of crutches for walking. With appropriate therapy, further improvement in his walking ability can be expected.

## Conclusions

The incidence of syphilis declined in high-income countries after World War II, as effective antimicrobial therapy, combined with expanded screening, diagnosis, and treatment programs became more readily available. In the last decade, however, rates have increased in the United States and Europe [1]. Syphilis is classified into three stages: primary; secondary or latent; and tertiary. Each stage is characterized by different symptoms and levels of infectivity. If untreated, tertiary syphilis can occur from 2 to 30 years after primary infection, with pockets of damage accumulating in various tissues, including the bones, skin, nervous tissue, heart, and arteries.

Tabes dorsalis, a unique manifestation of late tertiary neurosyphilis, arises in 2-9% of subjects with untreated syphilis between 3 and 50 years after primary infection. Neuropathic arthropathy occurs in 6-10% of these patients [8].

Syphilis may affect the natural history of HIV infection, and unusual clinical manifestations of syphilis and neurosyphilis may be more frequently seen in HIV-positive patients [9,10]. Although the relationship between these two diseases remains controversial, the course of syphilis seems to be more rapid and malignant in HIV-positive patients [9,10]. Clinically, HIV-syphilis co-infection seems to be associated with multiple or deeper chancres, overlap of features of primary and secondary syphilis, more rapid progression to tertiary syphilis, clinically important neurologic disease, and a shorter latency period than meningovascular syphilis.

Recently, a case neuropathic arthropathy resembling Charcot disease was described in a 73-year-old man without a history of HIV or HCV infection [2]. In this patient, progression of disease was less rapid and bone destruction less extensive than in our patient who also presented pain at the second visit, unlike the case described by Viens et al. [2]. The poor compliance of our patient, his reticence about his serostatus, was a confounding factor in this case which delayed establishing a correct diagnosis. HIV-HCV co-infection may likely have influenced the course of syphilis, leading to the need for surgery, which was avoided in the case Viens et al. described, although direct evidence for this hypothesis is lacking. The poor bone condition and the increased risk of infection moved us to prefer a Girdlestone's procedure over prosthetic arthroplasty as the surgical approach.

This arthroplasty technique entails resection of the femoral head with subsequent support of the trochanter body to the acetabular edge. Compared with other more invasive techniques, it has the advantage that it allows for a discrete recovery of joint function, while limiting the risk of infection, and provides for further joint reconstruction [11].

In the case presented, diagnosis was essentially based on exclusion methodology since it was not possible to perform DNA test and/or special staining. These analyses could likely provide useful information for a direct diagnosis of neuropathic syphilitic involvement.

In conclusion, due to the resurgence of syphilis, this disease should be considered as a possible etiology of neuropathic arthropathy when other infectious causes have been excluded, particularly when concomitant HIV or HIV-HCV infection is present.

## Competing interests

The authors declare that they have no competing interests.

## Authors' contributions

LD and EDV performed the microbiological analyses and assisted in writing the manuscript; MP and MB cared for the patient and assisted in writing the manuscript; LM performed the histological evaluations. All authors have read and approved the manuscript.

## Pre-publication history

The pre-publication history for this paper can be accessed here:

http://www.biomedcentral.com/1471-2334/11/159/prepub
